# Can simple measures from clinical practice serve as a proxy for sarcopenic obesity and identify mortality risk?

**DOI:** 10.1007/s40520-024-02866-9

**Published:** 2024-11-19

**Authors:** Valdete Regina Guandalini, Patrícia Silva Tofani, Sara Souza Lima, Letícia Coelho Silveira, Natália Cochar-Soares, Thais Barros Pereira da Silva, Thales Batista de Souza, Mariane Marques Luiz, Paula Camila Ramírez, Roberta de Oliveira Máximo, Andrew Steptoe, Cesar de Oliveira, Tiago da Silva Alexandre

**Affiliations:** 1 Nutrition and Health Postgraduate Program, Federal University of Vitoria, Vitoria, Brazil; 2 Gerontology Postgraduate Program, Federal University of Sao Carlos, Sao Carlos, Brazil; 3 Department of Physical Therapy, Federal University of Sergipe, Lagartos, Brazil; 4 Physical Therapy Postgraduate Program, Federal University of Sao Carlos, Sao Carlos, Brazil; 5 Escuela de Fisioterapia, Universidad Industrial de Santander, Bucaramanga, Colombia; 6 Department of Epidemiology and Public Health, University College London, London, UK; 7 Department of Gerontology, Federal University of São Carlos, Rodovia Washington Luís, km 235, São Carlos, São Paulo, 13565-905 Brazil

**Keywords:** Visceral obesity, Muscle mass, Sarcopenia, Older adults, Mortality

## Abstract

**Background:**

Sarcopenic obesity is a condition where loss of muscle mass occurs alongside fat gain, and it is considered a risk factor for mortality. However, the use of various definitions for this condition has led to conflicting results.

**Aim:**

To investigate whether the coexistence of low muscle mass and abdominal obesity, defined using two simple measures employed in clinical practice, is a risk factor for mortality in individuals aged 50 or older.

**Methods:**

A longitudinal study with a 14-year follow-up was conducted involving 5,440 participants of the *English Longitudinal Study of Ageing*. Abdominal obesity and low muscle mass were respectively defined based on high waist circumference and low skeletal muscle mass index (SMMI) determined by an equation. The sample was divided into four groups: non-low muscle mass/non-abdominal obesity (NLMM/NAO), non-low muscle mass/abdominal obesity (NLMM/AO), low muscle mass/non-abdominal obesity (LMM/NAO), and low muscle mass/abdominal obesity (LMM/AO). Cox regression models were used to estimate the mortality risk as a function of muscle mass and abdominal obesity status.

**Results:**

LMM/AO increased the risk of death by 83% (HR:1.83; 95%CI: 1.35–2.66) compared to those in the NLMM/NAO group. AO alone was not associated with a greater risk of mortality (HR:1.09; 95%CI: 0.93–1.27), whereas LMM alone increased the risk by 40% (HR:1.40; 95%CI:1.18–1.66).

**Conclusions:**

Identifying LMM/AO in individuals aged 50 or older can be crucial for predicting the risk of mortality. Simple and easily applicable measures can serve as a proxy for sarcopenic obesity and aid in implementing the necessary interventions.

**Supplementary Information:**

The online version contains supplementary material available at 10.1007/s40520-024-02866-9.

## Introduction

Sarcopenic obesity (SO) is characterised by excess adiposity and low muscle mass. Its prevalence increases with age [1, 2]. The European Society for Clinical Nutrition and Metabolism (ESPEN) and the European Association for the Study of Obesity (EASO) established diagnostic criteria for SO. Based on these criteria, SO is defined by a high percentage of body fat (% BF) associated with low muscle mass, both of which can be assessed by bioelectrical impedance analysis (BIA) or dual-energy X-ray absorptiometry (DXA) [3].

However, BIA or DXA are not widely available in clinical practice and have limitations that should be considered. BIA may underestimate the amount of body fat due to inaccuracies of the method, the presence of oedema, or the hydration status of the fat-free mass. Moreover, the examination requires complex protocols [4, 5]. Similarly, DXA cannot quantify intramuscular adipose tissue and may overestimate muscle mass in patients with extracellular fluid accumulation and underestimate fat mass. Therefore, when access to these devices is not possible, using anthropometric measurements is indicated as a viable, low-cost, widely available alternative [4, 5].

The coexistence of abdominal obesity, which is defined by high waist circumference (WC), and low muscle mass obtained by an anthropometric equation, could be considered a proxy for SO, as the investigation of this condition remains incipient in clinical practice due to different definitions and the lack of a universally accepted classification [5].

Prospective studies investigating the association between SO and mortality risk have presented conflicting results. A study involving Americans found no association between these variables [6]. In a study involving Swedish individuals aged 60 or older, a higher risk of mortality was found among women with SO but not men [7]. In these studies, SO was based on the percentage of body fat (%BF), body mass index (BMI), or waist circumference (WC), and sarcopenia was determined by the appendicular skeletal muscle mass index (ASMMI) obtained by BIA or DXA. Only one of the studies followed the criteria of the European Working Group on Sarcopenia in Older People (EWGSOP2) [8].

In three British cohort studies analysing individuals between 40 and 79 years of age, the mortality risk was only found in men with SO defined by WC and arm muscle circumference (AMC) in one of the studies [9]. In contrast, the other studies found no association between SO (defined by BMI and muscle strength) and a greater risk of mortality [10, 11]. An increase in the risk of mortality was found in Japanese American men between 71 and 93 years of age in the group with SO only when obesity was defined by WC [12].

The results of these studies reveal a need for more consensus on methods and classifications in diagnosing SO. A simple proxy for OS has not been determined by combining high WC and low skeletal muscle mass, as assessed by the equation proposed by Lee et al. [13]. This proposal could expand OS screening in individuals with different socioeconomic conditions and settings and health care services. Therefore, the present study hypothesises that the use of simple measures employed in clinical practice to define abdominal obesity by high WC and low muscle mass determined by the skeletal muscle mass index (SMMI) obtained using Lee’s equation is capable of identifying the risk of mortality in individuals 50 years of age or older.

## Methods

### Study design and participants

The data analysed in this study were extracted from the English Longitudinal Study of Ageing (ELSA). ELSA is a panel study started in 2002 with a representative sample of community-dwelling English men and women aged 50 or older [13]. Details on the ELSA methods can be found in a previous publication [14].

The analysis involved 5,440 participants aged ≥ 50 who participated in Wave 2 of ELSA (2004–2005). We examined overall mortality in a 14-year follow-up period (2018–2019). The Supplemental Material (Study population section) provides detailed information on the sample selection process and a sample selection flowchart.

### Abdominal obesity

Abdominal obesity (AO) was determined using waist circumference (WC). WC at baseline was measured at the midpoint between the last rib and the upper margin of the iliac crest. The measurement was taken twice at the end of the expiratory phase of the breathing cycle. The average of two valid measurements (or the closest measurements if three were made) was used for analysis. Abdominal obesity was recorded when WC was > 102 cm in men and > 88 cm in women [15].

### Muscle mass

Skeletal muscle mass (SMM) was estimated using Lee’s Eqs. [16, 17]. In a previous study, Veronese et al. and Spexoto et al. used this equation to estimate SMM to investigate the association between multimorbidity at baseline and the onset of sarcopenia over a 12-year follow-up and sarcopenia as a risk factor for mortality over a 14-year follow-up, respectively, in a large representative sample of English older adults [18]. The skeletal muscle mass index (SMMI) (kg/m^2^) was calculated by the ratio of SMM (kg) divided by the square of height in metres [19]. SMMI < 9.36 kg/m^2^ for men and < 6.73 kg/m^2^ for women (lowest 20th percentile of the population) was considered indicative of low muscle mass [18, 20]. All measures used to define and diagnose low muscle mass were performed at baseline.

### Low muscle mass/abdominal obesity (LMM/AO)

At baseline, ELSA participants were divided into four groups based on muscle mass and abdominal obesity status: non-low muscle mass/non-abdominal obesity (NLMM/NAO), non-low muscle mass/abdominal obesity (NLMM/AO), low muscle mass/non-abdominal obesity (LMM/NAO), and low muscle mass/abdominal obesity (LMM/AO).

### Mortality

Mortality data were obtained from the Office for National Statistics of England. All-cause mortality was investigated over a 14-year follow-up period for the present analyses.

### Covariates

Based on previous studies that investigated the association between SO and mortality [9, 11, 20, 21], we selected the following sociodemographic, behavioural, and clinical variables collected at baseline to be incorporated into the present analysis: sex, age (50–59, 60–69, 70–79, and 80 or more), race (white or non-white), total household wealth (in quintiles), marital status (with or without conjugal life), education level (0 to 11 years, 12 to 13 years, and > 13 years), smoking status (non-smokers, former smokers, and current smokers), and alcohol intake (never/rarely, frequently, daily, or not declared) [22, 23]. Physical activity level was investigated using three questions from the Physical Activity and Sedentary Behaviour Assessment Questionnaire (PASBAQ) validated by the Health Survey for England [24]: 1. Frequency of vigorous sports or activities: more than once a week, once a week, one to three times a month, hardly ever, or never?” 2. Frequency of moderate sports or activities: more than once a week, once a week, one to three times a month, hardly ever, or never?” 3. Frequency of light sports or activities: more than once a week, once a week, one to three times a month, hardly ever, or never?“ [25]. Moderate or vigorous physical activity at least once per week classified individuals as active. These individuals were united in one group due to the small number of participants in these categories. Only light activity at least once per week classified individuals as having low physical activity, and no weekly activity classified individuals as inactive [26].

Clinical conditions were recorded based on self-reports of a medical diagnosis of systemic arterial hypertension, diabetes, cancer, lung disease, heart disease, stroke, and metabolic syndrome. Detailed information on clinical conditions can be found in the Supplemental Material (Covariates section). Depressive symptoms were investigated using the eight-item Center for Epidemiological Studies-Depression Scale, for which a score of ≥ 4 was considered indicative of the presence of depressive symptoms [27] Memory status was assessed using the Word-List Learning Test, which ranges from 0 to 20 words, with higher scores indicating a better memory performance [28].

### Ethical considerations

All participants of the ELSA study signed a statement of informed consent, and all waves of the study received approval from the London Multicentre Research Ethics Committee [MREC/01/2/91]).

### Statistical analysis

The characteristics of the sample at baseline were expressed as mean, standard deviation, and proportion. Differences in baseline characteristics among the four analytical groups were assessed using the chi-square test and analysis of variance (ANOVA) with the Bonferroni post-hoc test. We examined all deaths that occurred in the 14-year follow-up period. Time for those who were deceased was calculated by the difference between the date of death (day/month/year) and the date of the baseline interview. Time for those who lived through the end of the follow-up period was calculated by the difference between the last data recorded (visit/interview) and the date of the baseline interview. Survival curves were plotted using the Kaplan–Meier method to explore the association between muscle mass and abdominal obesity status with mortality. Differences between curves were investigated using the log-rank test. The Cox regression model was run to explore the association between muscle mass and abdominal obesity status and mortality. Unadjusted and adjusted hazard ratios (HRs) were estimated with their respective 95% confidence intervals (CIs). The adjusted model was controlled for all sociodemographic, behavioural, and clinical variables. Furthermore, as caution should be exercised when using the Lee equation for individuals with BMI > 30 kg/m^2^, a sensitivity model was performed, including only participants with BMI ≤ 30 kg/m^2^. The Stata 16^®^ statistical package was used for all analyses, with a p-value < 0.05 considered indicative of statistical significance.

## Results

Among the 5,440 participants followed up for 14 years, 1,319 deaths were reported, corresponding to 24.2% of the sample. The prevalence of NLMM/AO, LMM/NAO, and LMM/AO was 49.8%, 19.0%, and 1.5%, respectively. The sociodemographic, behavioural, clinical, and anthropometric characteristics of the participants at baseline are displayed in Table 1. At baseline, the mean age was 65 years (± 9.2). The sample was predominantly composed of women (54.1%), individuals with a low education level (48.7%), those with a low physical activity level (94.5%), ex-smokers (48.9%), and frequent alcohol intake (41.5%). The most prevalent clinical conditions were arterial hypertension (41.0%), metabolic syndrome (27.7%), and heart disease (19.3%). Many participants were overweight (44.7%) (Table 1).

**Table 1:** Baseline sociodemographic, behavioural and clinical characteristics according to low muscle mass and abdominal obesity status in 5,440 participants of ELSA (2004)

	Total *n* = 5.440	NLMM/NAO *n* = 1777	NLMM/AO *n* = 2.627	LMM/NAO *n* = 955	LMM/AO *n* = 81
**Sociodemographic characteristics**					
Age, years (SD)	65.2 ± 9.2	62.4 ± 7.3	65.2 ± 8.7^a^	71.2 ± 9.2ª^,b^	80.8 ± 6.7ª^,b, c^
Age (%)					
50–59	32.6	31.4	33.2^a^	16.5ª^,b^	1.2ª^,b, c^
60–69	35.1	40.3	35.1^a^	27.9ª^,b^	4.9ª^,b, c^
70–79	23.2	15.6	25.0^a^	32.1ª^,b^	27.2ª^,b^
80 or more	9.1	2.3	6.8^a^	23.5ª^,b^	66.7ª^,b, c^
Sex (female), (%)	54.1	47.2	58.9	51.7	79.0
Marital status (without conjugal life), (%)	31.0	25.6	30.0ª	41.3^b^	64.2^a, b,c^
Total household wealth, (%)					
Lowest quintile	14.1	9.4	16.0^a^	16.8a	22.2^a^
2nd quintile	18.5	24.7	20.6^a^	21.6	19.8
3rd quintile	20.6	21.0	22.0	16.4^a, b^	16.0
4th quintile	22.1	15.6	20.1^a^	19.2a	21.0
Highest quintile	23.4	28.0	19.7^a^	25.2b	21.0
Not reported	1.3	1.2	1.6^a^	0.8^b^	-
Educational level, (%)					
0–11 years	48.7	39.6	53.0^a^	53.3^a^	58.0^a^
12–13 years	25.3	27.6	24.7	22.9	22.2
> 13 years	26.0	32.8	22.3^a^	23.8^a^	19.8^a^
Race (non-white), (%)	1.5	2.1	1.6ª	0.2ª^,b^	-
**Behavioural characteristics**					
Physical activity (%)					
Inactive	3.1	1.4	3.3 ^a^	4.8 ^a^	13.6 ^a, b,c^
Low	94.5	96.7	94.0	92.7^a^	84.0 ^a, b,c^
Moderate/vigorous	2.4	1.9	2.6	2.5	2.5
Smoking status, (%)					
Non-smoker	37.3	41.2	35.5^a^	35.6^a, b^	32.1
Former smoker	48.9	45.6	51.1	47.7 ^a, b^	60.5^a^
Smoker	13.8	13.2	13.4	16.6 ^a, b^	7.4
Alcohol intake, (%)					
Never/rarely	16.0	12.2	18.0	17.0^a, b^	22.2^a^
Frequently	41.5	41.4	42.7^a^	39.2	37.0
Daily	34.1	40.3	30.4^a^	33.7^a^	22.2^a^
Not declared	8.40	6.1	9.0^a^	10.2^a, b^	18.5^a, b^
**Clinical conditions (yes) (%)**					
Systemic arterial hypertension	41.0	32.4	48.9^a^	34.7^b^	48.1a
Diabetes	7.4	4.6	10.2 ^a^	4.5 ^a, b^	12.3 ^a, b,c^
Cancer	7.1	5.4	7.6^a^	8.7^a^	11.1
Lung disease	7.0	14.1	19.1^a^	19.8 ^a, b^	27.2^a^
Heart disease	19.3	16.3	19.8^b^	22.4^a, b^	32.1
Stroke	3.4	2.6	3.1	4.6	13.6 ^a, b,c^
Metabolic syndrome	27.7	12.0	45.8 ^a^	8.3 ^a, b^	16.0^b^
Depressive symptoms	13.1	10.4	14.6 ^a^	13.0	28.4 ^a, b,c^
Memory Score, points (SD)	10.3 ± 3.4	10.8 ± 3.2	10.2 ± 3.3^a^	9.5 ± 3.6ª^,b^	8.5 ± 3.4ª^,b^
**Anthropometry**					
Body mass index, (%)					
Eutrophic	27.8	31.1	1.6	92.0	49.4
Overweight	44.7	65.8	43.5	8.0	50.6
Obesity	27.5	3.1	54.9	-	-
BMI, kg/m^2^ (SD)	27.8 ± 0.6	26.0 ± 2.1	31.1 ± 4.1^a^	22.4 ± 1.8 ^a, b^	24.9 ± 1.4 ^a, b,c^
Waist circumference, cm (SD)	95.4 ± 12.7	89.2 ± 8.3	104.0 ± 10.1^a^	83.0 ± 8.5 ^a, b^	95.0 ± 6.1 ^a, b,c^
SMMI kg/m^2^ (SD)	8.9 ± 1.7	8.8 ± 1.4	9.6 ± 1.6ª	7.5 ± 1.4ª^,b^	7.0 ± 1.2 ^a, b,c^

Note Data is expressed as the proportion, mean, and standard deviation. Abbreviations: SD = standard deviation. NLMM//NAO: Non-low muscle mass/non-abdominal obesity; NLMM/AO: Non-low muscle mass/abdominal obesity; LMM/NAO: Low muscle mass/non-abdominal obesity; LMM/AO: Low muscle mass/abdominal obesity. BMI: Body mass index; SMMI: skeletal muscle mass index. ^a^ Significantly different from NLMM/NAO; ^b^ Significantly different from NLMM/AO; ^c^ Significantly different from LMM/NAO (*p <* 0.05)

The participants in the LMM/AO category were older, mostly women, fewer had a marital life, were more physically inactive, had greater frequencies of diabetes mellitus and stroke, had more depressive symptoms, and had a lower mean SMMI than participants in the NLMM/NAO, NLMM/AO, and LMM/NAO categories. BMI in the LMM/AO group was lower than that in the NLMM/AO and NLMM/NAO groups but higher than that of the LMM/NAO group. WC in the LMM/AO group was lower than that in the NLMM/AO group but higher than in the LMM/NAO and NLMM /NAO groups (Table 1).

The participants in the LMM/NAO group were older, predominantly women, more frequently categorised as white, smoked more, had lower rates of diabetes mellitus and metabolic syndrome, higher rates of lung and heart disease, a lower memory score, lower BMI, lower WC, and lower SMMI than the participants in the NLMM/NAO group (Table 1).

The participants in the NLMM/AO group were older, fewer had a marital life, had a lower income and educational level, were more frequently categorised as white, were more physically inactive, smoked less, had higher rates of hypertension, diabetes mellitus, cancer, lung disease, heart disease, and metabolic syndrome, and higher BMI, WC, and SMMI than the participants in the NLMM/NAO group (Table 1).

**Fig. 1:**
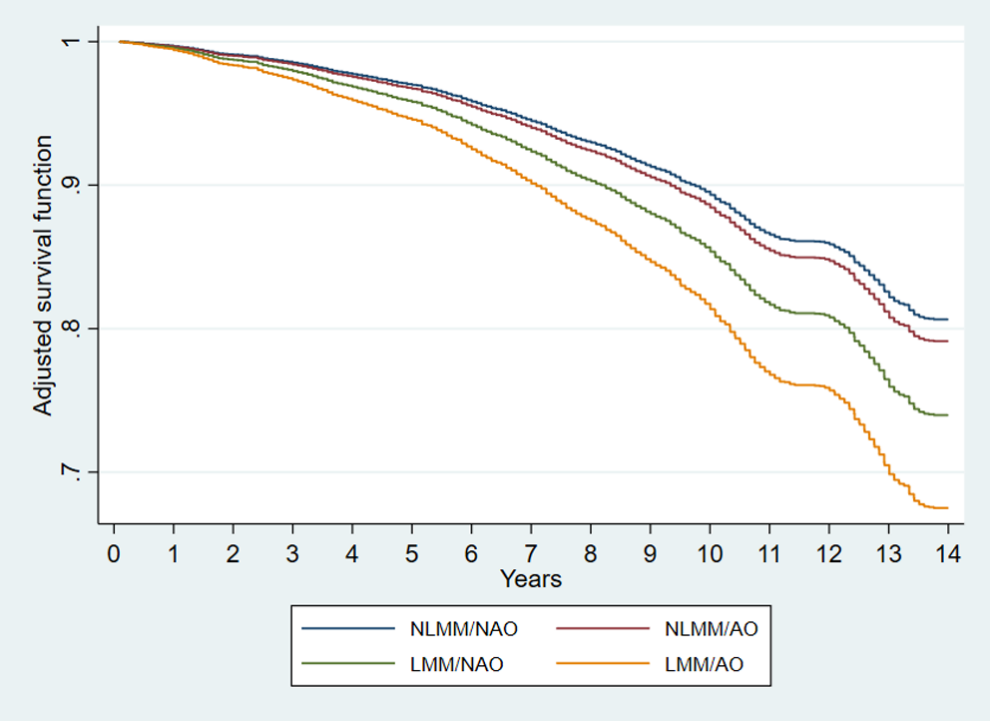
Survival analysis of low muscle mass/abdominal obesity status based on the final Cox proporcional hazards model. NLMM/NAO: non-low muscle mass/non-abdominal obesity; NLMM/AO: non-low muscle mass/abdominal obesity; LMM/NAO: low muscle mass/non-abdominal obesity; LMM/AO low muscle mass/abdominal obesity

Figure 1 displays the survival curve for abdominal obesity and LMM status over the 14-year follow-up period. The Cox model adjusted by sociodemographic, behavioural, and clinical variables revealed that the risk of mortality in the participants classified with LMM/NAO was 40% higher (HR: 1.40; 95% CI: 1.18–1.66) compared to those in the NLMM/NAO group. The risk of mortality in the participants classified with LMM/AO was 83% (HR: 1.83; 95% CI: 1.35–2.66) higher compared to those in the NLMM/NAO group, which is double the risk compared to those LMM/NAO. No increased mortality risk was found among participants in the NLMM/AO group compared to those in the NLMM/NAO group (HR: 1.09; 95% CI: 0.93–1,27) (Table 2).

**Table 2:** The final Cox Proportional Hazard Model predicting mortality in a 14-year follow-up among 5,440 participants from the ELSA Study (2004–2018)

	HR (95% CI)	*p* value
Non-low muscle mass/non-abdominal obesity	1,00	
Non-low muscle mass/abdominal obesity	1.09 (0.93–1.27)	0.285
Low muscle mass/non-abdominal obesity	1.40 (1.18–1.66)	< 0.001
Low muscle mass/abdominal obesity	1.83 (1.35–2.66)	< 0.001

HR: Hazard ratio; CI: Confidence interval. The adjusted model is comprehensive, considering sex, age, total household wealth, marital status, smoking status, alcohol intake, physical activity level, systemic arterial hypertension, diabetes, cancer, lung disease, heart disease, stroke, metabolic syndrome, depressive symptoms, memory score and race

The sensitivity analysis, including only participants with BMI ≤ 30 kg/m^2,^ demonstrated that participants with LMM/NAO had a 34% higher risk of mortality (HR: 1.34; 95% CI: 1.13–1.60) than those with NLMM/NAO. The mortality risk in the participants classified with LMM/AO was 66% (HR: 1.66; 95% CI: 1.21–2.27) higher than those in the NLMM/NAO group. No increased mortality risk was found among participants in the NLMM/AO group compared to those in the NLMM/NAO group (HR: 0.96; 95% CI: 0.80–1.15) (Table S1).

## Discussion

The main findings of the present study demonstrate that individuals with LMM/AO were at greater risk of mortality in a 14-year follow-up period compared to individuals with neither of these conditions at baseline. Individuals with LMM alone (LMM/NAO group) were also at greater mortality risk than those in the NLMM/NAO group but with half the effect size. Lastly, abdominal obesity alone did not indicate a greater risk of mortality.

Previous longitudinal studies found different results when investigating the association between low muscle mass (sarcopenia), obesity, and an increased risk of mortality among older adults [6, 7, 9, 12]. This divergence may be explained by differences in the methods employed and different cut-off points used to define and diagnose sarcopenia and obesity [29, 30].

Among the studies cited, obesity was determined based on %BF, BMI, and WC, and SMM was determined using either BIA or DXA [6, 7, 9, 12]. A Swedish study that followed 521 older adults (mean age: 75 years) for ten years found that SO was associated with an increased risk of mortality when obesity was defined by WC and/or BMI in models adjusted by comorbidities and smoking but not when obesity was classified exclusively based on %BF [7]. Batsis et al. [6] followed 4,652 older American adults (mean age: 70 years) for 14 years. Also, they did not find SO defined by %BF and BIA to be associated with an increased risk of mortality in a model adjusted by sociodemographic, behavioural, and clinical characteristics as well as limited mobility [5]. In contrast, other studies found an association between SO defined by WC and SMM determined by anthropometric measures and an increased risk of mortality [9, 12] in models controlled by sociodemographic and behavioural variables [9, 12], cardiovascular markers [9], and cognitive performance [12].

Using an anthropometric equation to determine SMM combined with WC to diagnose abdominal obesity, the mortality risk was 83% in the group with LMM/AO compared to the NLMM/NAO group. This finding is similar to that reported for older Swedish adults in the study by Von-Berens et al., who classified SO by WC (≥ 88 cm for women and ≥ 102 cm for men) or BMI (≥ 30 kg/m^2^) and determined the SMMI through BIA (≤ 8.5 kg/m^2^ for men and ≤ 5.75 kg/m^2^ for women) and found that the risk of mortality was 123% higher among individuals with SO compared to those in the NO/NS category [7]. Similarly, Atkins et al. [9] followed 4,111 English men with a mean age of 70 years, defining SO by WC (≥ 102 cm) and arm muscle circumference (≤ 25.9 cm). They found that the risk of mortality was 44.0% higher in individuals with SO compared to those in the NO/NS category. Sanada et al. [12] found a 19% higher risk of mortality in individuals with SO based on WC (≥ 85.0 cm) and the SMMI (< 7.77 kg/m^2^ determined using the anthropometric equation) in a sample of 2,309 Japanese men with a mean age of 77 years. However, Batsis et al. [6] used %BF (> 38% for women and > 27% for men) and SMMI determined by BIA (≤ 8.5 kg/m^2^ for men and ≤ 5.75 kg/m^2^ for women) to diagnose SO in older American adults and did not find an association between SO and an increased risk of mortality. In contrast, Campos et al. investigated the association between SO and mortality risk in a cohort of older Brazilians, classifying sarcopenia based on muscle strength and appendicular lean muscle index (ALMI) obtained by DXA (grip strength < 16 kg and ALMI < 6.0 kg/m² for women; grip strength < 27 kg and ALMI < 7.0 kg/m² for men) and obesity based on %BF (≥ 38% for women and 27% for men). In the study, only the group with sarcopenia had an increased risk of mortality when compared to the reference group. The groups with SO and obesity alone were not associated with mortality risk in the final models.

One of the hypotheses that may explain these conflicting results is the inability of %BF and BMI to identify body fat distribution, especially abdominal fat [31, 32]. This is important, as the ageing process is accompanied by the accumulation of abdominal fat and the loss of muscle mass, which are closely associated with mortality [12]. WC is the best indicator of obesity in older people, as it reflects the accumulation of abdominal fat independently of less mass and subcutaneous fat [12, 33]. Regarding the assessment of muscle mass by BIA, a systematic review demonstrated that this equipment is not recommended for identifying sarcopenia in individuals aged 70 or older, as it may overestimate or underestimate SMM [34].

Therefore, the present proposal to use simple measures such as WC to define abdominal obesity and an equation composed of routinely available information in clinical practice to define SMM achieved similar results to those described in previous studies. This is particularly important, as WC and equation are affordable and practical, while DXA and BIA are costly, require preparation and scheduling, and are inaccessible to many healthcare services.

The mechanism that explains the greater risk in individuals with LMM/AO is the synergic, bidirectional, amplified effect of low SMM and obesity on the metabolism and, consequently, the incidence of diseases and the risk of death [35–37]. Low SMM can reduce resting metabolic rate and energy expenditure, which, in turn, induces gains in body fat and weight. Similarly, obesity can favour the development and progression of low SMM due to inflamed adipose tissue, which has adverse effects on skeletal muscle, including insulin resistance, a reduction in fat oxidation, mitochondrial dysfunction, intramyocellular lipid deposition, and protein degradation, leading to muscle impairment and a decrease in muscle regeneration capacity [36, 37].

Low muscle mass alone was also associated with an increased risk of mortality, which agrees with results described in previous studies [6, 9, 12, 38] and systematic reviews with meta-analyses [39, 40]. Sarcopenia has been associated with mortality irrespective of the population, definition, and follow-up period [40]. The mechanisms behind this association have not yet been clarified, and several conditions exert an influence, such as chronic diseases, malnutrition, hospitalisations, inadequate living habits and diet, the ageing process itself, as well as falls and fractures, which occur as a consequence of muscle weakness [39, 40]. Furthermore, sarcopenia is related to hormonal, genetic, inflammatory, and behavioural factors [41], which impedes attributing a single cause to this association.

In the present study, abdominal obesity alone was not associated with an increased mortality risk. Although controversial, this result has been described in previous studies [12, 42, 43] and has been denominated the ‘obesity paradox’, specifically in older people [32, 44, 45]. Even in chronic diseases, excess weight and obesity in older people can be considered a sign of robustness and more significant body reserves for facing such diseases [43].

Regarding BMI and WC, participants with LMM/AO had a lower mean BMI than those with NLMM/NAO and NLMM/AO and mean WC was lower in those with LMM/AO than those with NLMM/AO. Such differences may be explained by how changes in body composition occur throughout ageing. In terms of physiology, a progressive muscle mass reduction occurs in the third decade of life. Fat mass, represented by the increase in visceral adipose tissue and intramuscular fat infiltration, increases until 70–75 years, diminishing after that. Thus, there is a more pronounced loss of total mass, muscle mass, and abdominal adiposity among older people, which explains the lower BMI and WC ​​in participants with LMM/AO compared to those with NLMM/AO [46, 47].

According to guidelines for the definition and diagnostic criteria of SO, obesity can be defined by BMI or WC. In contrast, sarcopenia can be determined by low muscle function and low skeletal muscle mass [3]. However, we did not include low muscle strength in diagnosing sarcopenia [10, 11] to obtain a viable and accessible alternative to be used by health services. By combining obesity with low muscle strength, we would be analysing the condition of dynapenic obesity, which has been widely explored by our research group in previous studies and associated with the risk of functional decline [22], a decline in physical performance [23], mortality [48], cardiovascular mortality [49], and metabolic syndrome [50].

This study has some solid points and limitations that should be considered. Among the strong points, this is the first study to use WC and the equation proposed by Lee et al. to define low muscle mass and abdominal obesity, obtaining expressive results concerning the risk of mortality, which demonstrates the advantage of this equation for the determination of SMM over DXA and BIA, such as the low cost, practicality, and ease of use [3, 8, 30, 51]. Moreover, this study was conducted with a representative sample of male and female community-dwelling English people aged 50 or older, enabling the early detection of the relationship between LMM/AO and the risk of mortality over a 14-year follow-up period with a single way of defining LMM/AO and the use of a model controlled by a large number of recognised risk factors of mortality.

This study’s limitations include the fact that muscle mass was determined using an equation based on anthropometric measures. However, this equation has been validated using established methods, providing similar results to those obtained using magnetic resonance imaging and DXA, which are expensive resources that are not widely available and are highly impractical for use in epidemiological studies. Losses to follow-up constitute another limitation but are inherent to longitudinal studies involving older people. However, this is the first longitudinal study with a large sample size to analyse the association between LMM/AO and the risk of mortality, and it did not use muscle strength to determine sarcopenia. The third limitation of this study is related to the absence of information on food intake, which began to be collected only in Wave 9 of the ELSA study. However, this variable has also only been included in some previous studies.

## Conclusion

LMM/AO, measured by waist circumference and an equation for estimating skeletal muscle mass, is a risk factor for mortality in individuals aged 50 or older. Therefore, these two simple measures, which are easy to apply in different contexts of clinical practice, can serve as a proxy for sarcopenic obesity, enabling necessary interventions to be implemented quickly in older people at risk of death.

## Electronic supplementary material

Below is the link to the electronic supplementary material.

Supplementary Material 1

## Data Availability

No datasets were generated or analysed during the current study.
