# Supplementary material for: Can simple measures from clinical practice serve as a proxy for sarcopenic obesity and identify mortality risk?
Source: Aging Clin Exp Res. 2024 Nov 19;36(1):222. doi: 10.1007/s40520-024-02866-9 (PMC11573834; doi:10.1007/s40520-024-02866-9)
Supplement: Supplementary file 1 — Supplementary Material 1 [file 40520_2024_2866_MOESM1_ESM.docx]

**Title:**

**Can Simple Measures from Clinical Practice Serve as a Proxy for Sarcopenic Obesity and Identify Mortality Risk?**

**SUPPLEMENTARY MATERIAL**

1. Supplementary Methods
2. Supplementary References

**1. Supplementary Methods**

**Study population**

ELSA is a panel study that began in 2002 with a representative sample of English adults aged 50 and older. ELSA recruits participants using multistage stratified probability sampling with postcode sectors selected in the first stage and household addresses specified in the second stage.

ELSA interviews occur every two years, and questionnaires are administered at the participants’ homes. Health examinations, blood collection for the determination of biochemical measures, and physical performance tests occur every four years with a visit from a nurse to the participants’ homes. Nurse visits were carried out for the first time in 2004/5 (Wave 2), which corresponded to the baseline of this analysis, as it was the first time data on obesity and anthropometric variables were collected. Wave 2 comprised 8,780 participants. For this analysis, 62 malnourished participants were excluded, as malnutrition is associated with an increased mortality risk (1).

Participants in Wave 2

n = 8,718

Exclusion due to missing data (n = 3,278)

- Missing in waist circumference and muscle mass (n=1,658)
- Missing in covariates (n=1,620)

Final sample

n= 5,440

Figure 1. Sample selection flowchart.

Detailed descriptions of the study, sampling procedures, and data collection have been published previously (2).

**Covariates**

Based on previous studies investigating the association between sarcopenic obesity and mortality, we selected sociodemographic, behavioural, and clinical variables collected at baseline to be incorporated in the present analysis. The covariates constitute a broad spectrum of mortality-related factors (3–8).

The sociodemographic variables were sex (men = 0; women = 1), marital status (with = 0 or without = 1 conjugal life), total household wealth (including financial, housing, and physical wealth, such as jewellery and artwork, classified in quintiles [5th quintile (top 20%) = 0; 4th quintile = 1; 3rd quintile = 2; 2nd quintile = 3, and 1st quintile (lowest 20%) = 4], and schooling (0 to 11 years = 2, 12 to 13 years = 1, and > 13 years = 0) based on the English educational system (9).

The behavioural characteristics were smoking status (non-smoker = 0, former smoker =1, or smoker =2) and weekly frequency of alcohol intake: “never or rarely” (≤ once per week = 0), “frequently” (two to six times per week = 1), “daily” (seven times per week = 2), or “not declared” = 3 (10–12). Physical activity level was assessed using three questions from the Physical Activity and Sedentary Behaviour Assessment Questionnaire (PASBAQ) validated by the Health Survey for England (13). The participants reported the frequency of vigorous (e.g., running/jogging, swimming, cycling, aerobics/gym workout, tennis, digging with a spade), moderate (gardening, cleaning the car, walking at a moderate pace, dancing), or light (laundry, home repairs) physical activity using cue cards with different activities to help them interpret different intensities. Physical activity was classified as moderate or vigorous = 0 (moderate or vigorous activity at least once per week) or low = 1 (only light activity at least once per week); individuals with no weekly activity were classified as inactive = 2 (14,15).

Clinical conditions were recorded based on self-reports of a medical diagnosis of systemic arterial hypertension, diabetes, cancer, lung disease, heart disease, and stroke (no = 0; yes = 1). Body mass index (BMI) was estimated using weight in kilograms divided by the square of height in meters (kg/m²). The participants were classified as being in the ideal range (≥ 18.5 and < 25.0 kg/m² = 0), underweight (< 18.5 kg/m² = 1), overweight (≥ 25.0 and < 30.0 kg/m² = 2) or with obesity (≥ 30 kg/m² = 3) (16).

Metabolic syndrome (MetS) was defined based on the recommendations of the consensus (IDF and NHLBI) and self-reports of the use of medications (17). Individuals with at least three of the following criteria were considered as having MetS: hypertriglyceridemia (fasting triglycerides ≥ 150 mg/ dl or use of omega-3 and/or fibrates and/or nicotinic acid); hyperglycaemia (fasting glucose ≥ 100 mg/dl or use of an oral antihyperglycemic agent and/or insulin); low HDL cholesterol (< 50 mg/dl for women and < 40 mg/dl for men or use of nicotinic acid to increase HDL cholesterol and/or fibrates); arterial hypertension (resting systolic pressure ≥ 130 mmHg and/or resting diastolic pressure ≥ 85 mmHg or use of antihypertensive agent). The obesity measure was modified from the original definition to avoid collinearity, as abdominal obesity would constitute the study exposure. Therefore, for the definition of MetS in this study, obesity was defined based on body mass index (BMI), a simple, non-invasive approach widely used in clinical practice and was used to define MetS in a previous study by our research group (18).

**Table S1** Sensitivity Analysis. Final Cox proportional hazard model predicting mortality in 14-year follow-up among 3,943 non-obese (BMI ≥ 30 kg/m²) participants from the ELSA Study (2004-2018).

|  | **HR (95% CI)** | **p value** |
| --- | --- | --- |
| Non-low muscle mass/non-abdominal obesity | 1,00 |  |
| Non-low muscle mass/abdominal obesity | 0.96 (0.80 – 1.15) | 0.652 |
| Low muscle mass/non-abdominal obesity | 1.34 (1.13 – 1.60) | <0.001 |
| Low muscle mass/abdominal obesity | 1.66 (1.21 – 2.27) | <0.001 |

HR: hazard ratio; CI: confidence interval. Adjusted model for sex, age, total household wealth, marital status, smoking status, alcohol intake, physical activity level, systemic arterial hypertension, diabetes, cancer, lung disease, heart disease, stroke, metabolic syndrome, depressive symptoms, memory score and race.

**2. Supplementary References**

1. Söderström L, Rosenblad A. Long-term association between malnutrition and all-cause mortality among older adults: A 10-year follow-up study. Clinical Nutrition. 2023 Dec;42(12):2554–61.

2. Steptoe A, Breeze E, Banks J, Nazroo J. Cohort Profile: The English Longitudinal Study of Ageing. Int J Epidemiol. 2013 Dec 1;42(6):1640–8.

3. Bernabe-Ortiz A, Carrillo-Larco RM, Gilman RH, Smeeth L, Checkley W, Miranda JJ. Skeletal muscle mass and all-cause mortality: Findings from the CRONICAS cohort study. Tropical Medicine and International Health. 2023 Feb 1;28(2):107–15.

4. Atkins JL, Whincup PH, Morris RW, Lennon LT, Papacosta O, Wannamethee SG. Sarcopenic Obesity and Risk of Cardiovascular Disease and Mortality: A Population‐Based Cohort Study of Older Men. J Am Geriatr Soc. 2014 Feb 15;62(2):253–60.

5. Batsis JA, Mackenzie TA, Barre LK, Lopez-Jimenez F, Bartels SJ. Sarcopenia, sarcopenic obesity and mortality in older adults: Results from the National Health and Nutrition Examination Survey III. Eur J Clin Nutr. 2014 Sep 1;68(9):1001–7.

6. Farmer RE, Mathur R, Schmidt AF, Bhaskaran K, Fatemifar G, Eastwood S V., et al. Associations Between Measures of Sarcopenic Obesity and Risk of Cardiovascular Disease and Mortality: A Cohort Study and Mendelian Randomization Analysis Using the UK Biobank. J Am Heart Assoc. 2019 Jul 2;8(13).

7. von Berens, Obling SR, Nydahl M, Koochek A, Lissner L, Skoog I, et al. Sarcopenic obesity and associations with mortality in older women and men – A prospective observational study. BMC Geriatr. 2020 Jun 9;20(1):1–10.

8. Sanada K, Chen R, Willcox B, Ohara T, Wen A, Takenaka C, et al. Association of sarcopenic obesity predicted by anthropometric measurements and 24-y all-cause mortality in elderly men: The Kuakini Honolulu Heart Program. Nutrition. 2018 Feb 1;46:97–102.

9. Alexandre T da S, Scholes S, Santos JLF, de Oliveira C. Dynapenic Abdominal Obesity as a Risk Factor for Worse Trajectories of ADL Disability Among Older Adults: The ELSA Cohort Study. The Journals of Gerontology: Series A. 2019 Jun 18;74(7):1112–8.

10. Spexoto MCB, Ramírez PC, De Oliveira Máximo R, Steptoe A, De Oliveira C, Alexandre TDS. European Working Group on Sarcopenia in Older People 2010 (EWGSOP1) and 2019 (EWGSOP2) criteria or slowness: Which is the best predictor of mortality risk in older adults? Age Ageing. 2022 Jul 1;51(7).

11. Máximo R de O, de Oliveira DC, Ramirez PC, Luiz MM, de Souza AF, Delinocente MLB, et al. Combination of dynapenia and abdominal obesity affects long-term physical performance trajectories in older adults: sex differences. Am J Clin Nutr. 2022 May;115(5):1290–9.

12. Ramírez PC, de Oliveira DC, de Oliveira Máximo R, de Souza AF, Luiz MM, Delinocente MLB, et al. Is dynapenic abdominal obesity a risk factor for cardiovascular mortality? A competing risk analysis. Age Ageing. 2023 Jan 8;52(1).

13. Joint Health Surveys Unit NCSR and UCLRD of E and PH. The Health Survey for England Physical Activity Validation Study: Substantive Report. NHS Information Centre for Health and Social Care: Leeds, UK. 2008.

14. Scholes S, Coombs N, Pedisic Z, Mindell JS, Bauman A, Rowlands A V., et al. Age- and sex-specific criterion validity of the health survey for England physical activity and sedentary behavior assessment questionnaire as compared with accelerometry. Am J Epidemiol. 2014 Jun 15;179(12):1493–502.

15. Craig R, Mindell J, Hirani V. Physical activity and fitness Health Survey for England 2008 2 A survey carried out on behalf of The NHS Information Centre. 2009.

16. Obesity: preventing and managing the global epidemic. Report of a WHO consultation. World Health Organ Tech Rep Ser. 2000;894:i–xii, 1–253.

17. Alexandre T da S, Aubertin-Leheudre M, Carvalho LP, Máximo R de O, Corona LP, Brito TRP de, et al. Dynapenic obesity as an associated factor to lipid and glucose metabolism disorders and metabolic syndrome in older adults – Findings from SABE Study. Clinical Nutrition. 2018 Aug;37(4):1360–6.

18. Ramírez PC, de Oliveira Máximo R, Capra de Oliveira D, de Souza AF, Luiz MM, Delinocente MLB, et al. Dynapenic Abdominal Obesity as a Risk Factor for Metabolic Syndrome in Individual 50 Years of Age or Older: English Longitudinal Study of Ageing. J Nutr Health Aging. 2023 Nov 30;
